# Dynamic Emotional Faces Generalise Better to a New Expression but not to a New View

**DOI:** 10.1038/srep31001

**Published:** 2016-08-08

**Authors:** Chang Hong Liu, Wenfeng Chen, James Ward, Nozomi Takahashi

**Affiliations:** 1 Department of Psychology, Faculty of Science and Technology Bournemouth University, Talbot Campus Fern Barrow Poole, Dorset, BH12 5BB, United Kingdom; 2 State Key Laboratory of Brain and Cognitive Science, Institute of Psychology, Chinese Academy of Sciences, 16 Lincui Road, Chaoyang District, Beijing 100101, China; 3 Department of Computer Science, University of Hull, Cottingham Road, Hull, HU6 7RX, United Kingdom; 4 Department of Psychology, Graduate School of Literature and Social Science Nihon University, 3-25-40, Setagaya-ku, Sakurajosui Tokyo 156-8550, Japan.

## Abstract

Prior research based on static images has found limited improvement for recognising previously learnt faces in a new expression after several different facial expressions of these faces had been shown during the learning session. We investigated whether non-rigid motion of facial expression facilitates the learning process. In Experiment 1, participants remembered faces that were either presented in short video clips or still images. To assess the effect of exposure to expression variation, each face was either learnt through a single expression or three different expressions. Experiment 2 examined whether learning faces in video clips could generalise more effectively to a new view. The results show that faces learnt from video clips generalised effectively to a new expression with exposure to a single expression, whereas faces learnt from stills showed poorer generalisation with exposure to either single or three expressions. However, although superior recognition performance was demonstrated for faces learnt through video clips, dynamic facial expression did not create better transfer of learning to faces tested in a new view. The data thus fail to support the hypothesis that non-rigid motion enhances viewpoint invariance. These findings reveal both benefits and limitations of exposures to moving expressions for expression-invariant face recognition.

A change of facial expression from training to test is known to impair recognition of unfamiliar faces^1^. However, relative to effects of viewpoint and illumination on face recognition, little research has investigated how human observers handle image variation due to facial expression. Liu, Chen, and Ward (2015) have found recently that repeated exposures to several facial expressions could facilitate generalisation of training to a new expression^2^. In their study, participants either learnt each face with three randomly chosen expressions (e.g., sadness, surprise, and disgust) or a single expression (e.g., sadness). Their key finding was that learning three expressions of a face generalises better to the same face with a new expression (e.g., anger). However, their study also demonstrated that the improvement was small and restricted. Even after several exposures to each expression, face recognition performance remained vulnerable when the studied face was switched to a new expression in the test session.

An important limitation of Liu *et al*. (2015) was that their experiments were based on still face images^2^. Although people do learn new faces from photographs and still images in prints and from the Internet, faces are often learnt through video images or direct interaction with them in reality. Research has shown that people are often more effective in learning and recognising dynamic faces^3^,^4^,^5^ (see the references for reviews). There is also evidence that motion information alone allows for identity recognition^6^. Hence dynamic facial expression could offer a more effective route to expression-invariant recognition. A main purpose of the present study was to assess whether exposure to moving facial expressions creates better generalisation of learnt faces to a new expression. Because the sequence of a video can provide richer information of image variation created by non-rigid facial movement, each expression in motion should allow formation of a more robust representation. Although little is known about how exposure to a dynamic facial expression improves face recognition, recent research has shown that learning multiple still face images can facilitate face matching^7^,^8^,^9^ and recognition memory^2^. These studies would predict an advantage for faces learnt from multiple frames in a video clip because the dynamic information is conveyed by a sequence of multiple images. The advantage of learning multiple images of a face may also be attributed to averaging. Research has shown that averaging across multiple images of a face can create a robust representation against a range of image variations including expression variation^10^. Averaging may also employ information contained in moving images.

Another advantage of learning a face with non-rigid motion is that subsequent recognition appears to be more resilient to viewpoint variation. Early evidence for this can be found in Thornton and Kourtzi^11^, who showed that a short video sequence aided matching performance when the sequentially displayed test face differed in expression or viewpoint. Watson, Johnston, Hill, and Troje^12^ later showed that this advantage can be demonstrated when an identity judgement of two sequentially displayed face stimuli was based on non-rigid motion alone, where non-rigid motion captured from different identities was applied to a single 3D model. These studies, as pointed out by Thornton and Kourtzi^11^, mainly address face recognition in the working memory because they used a matching task. It is, therefore, important to know whether the effect of non-rigid motion applies to long-term memory because creating long-term representations of studied faces is often the ultimate goal of face learning.

We therefore investigated two questions about the potential advantages of learning faces with dynamic facial expressions. First, we tested the prediction that studying expressions of a face with motion information facilitates transfer of the studied face to a new expression. Second, we assessed the hypothesis that the viewpoint invariant effect created by non-rigid facial motion can extend to long-term recognition memory. We conducted two experiments; each addressed one of these questions.

Both experiments in this study employed the standard old/new recognition task to probe the effects of the experimental manipulations on long-term face memory. Participants were instructed to remember faces in a learning session. Their memory of the learnt faces was later assessed in a test session.

Experiment 1 tested the prediction that learning dynamic facial expressions facilitates better transfer to a new emotional expression relative to learning static images of facial expressions. It also assessed whether learning three expressions is equally beneficial for the transfer in the dynamic and static expression conditions.

The potential advantage of learning faces with a dynamic facial expression was assessed only in frontal-view face stimuli in Experiment 1, where the matching and recognition tasks required judging facial identity in the same view. In Experiment 2, we assessed whether faces showing dynamic expressions could generalise better to a new view. If non-rigid motion of a facial expression creates viewpoint invariant representation, we would expect little detrimental effect of viewpoint change for identity recognition. We also investigated whether moving faces in a three-quarter view are more robust against viewpoint change than a frontal view. In Experiment 2, participants learnt faces either in a frontal or a three-quarter view. In the test session, the learnt faces were either shown in the same view or a different view. As in Experiment 1, the expression at test was always different from learning.

## Results

All statistical tests in this study are two tailed. The Cox-Small test was used to assess the multivariate normality of the results in Experiment 1 and the test session results of Experiment 2. The Shapiro-Wilk’s test was used to assess the univariate normality of the learning session results in Experiment 2.

### Experiment 1

#### Learning session results.

**Figure 1:**
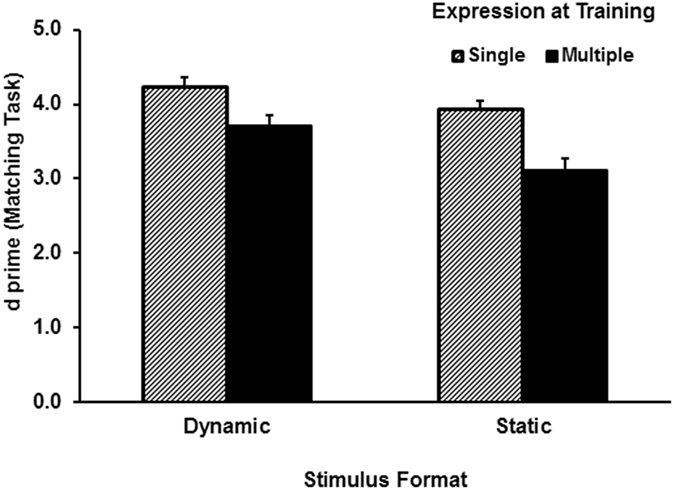
Face matching *d′* results as a function of stimulus format and exposure in the learning session of Experiment 1. Error bars represent one standard error above the means. N = 78.

Figure 1 shows the mean *d′* results for the sequential matching task. As *d′* data violated the assumption of multivariate normality (*p’*s ≤ 0.005), we used nonparametric tests. Wilcoxon tests for related samples showed that performance was superior when faces were matched in a single expression relative to three expressions. This was true for both the dynamic condition, Ζ = −2.99, *p* < 0.01, and the static condition, Ζ = −3.23, *p* < 0.001. Mann-Whitney tests for independent samples showed that dynamic faces created better matching performance than static faces in the multiple expression condition, Ζ *=* −2.56*, p* *<* 0.01. The same effect in the single expression condition was marginally significant, Ζ = −1.87, *p* = 0.062.

The criterion results are shown in Table 1. Criterion data were normally distributed (*p* = 0.684). ANOVA on the criterion data showed a significant main effect of exposure, where criterion for the multiple-expression condition was more conservative than for the single-expression condition, *F* (1, 76) = 8.15, partial *η*^2^ = 10, *p* < 0.01. The criterion was more conservative for matching dynamic faces than for static faces, *F* (1, 76) = 4.08, partial *η*^2^ = 0.05, *p* < 0.05. The interaction between the two factors was also significant, *F* (1, 76) = 5.73, partial *η*^2^ = 0.07, *p* < 0.02. Further analysis of the interaction using paired samples t-tests showed that whereas results for multiple- and single-expression conditions in the dynamic-face group were comparable, *t* (39) = 0.58, *p* = 0.57, criterion for the single-expression condition was more liberal than for the multiple-expression condition in the static-face group, *t* (37) = 3.26, *p* < 0.01.

#### Test session results

**Figure 2:**
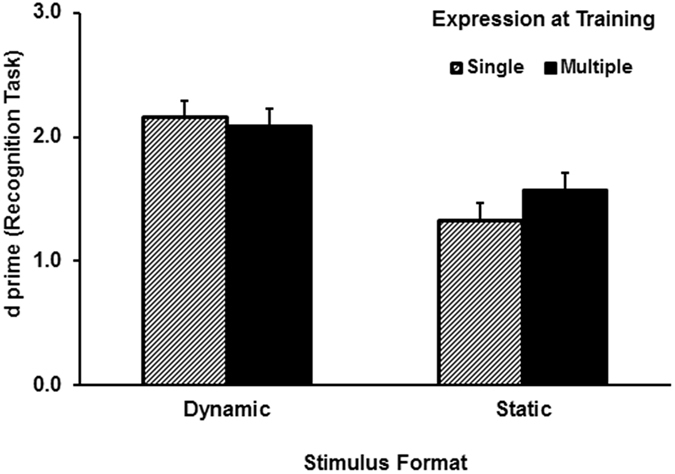
Face recognition *d′* results of as a function of stimulus format and exposure in the test session of Experiment 1. Error bars represent one standard error above the means. N = 78.

Both the *d′* and *c* data were normally distributed (*p’*s > 0.05). Figure 2 shows the mean *d′* results of the recognition test. ANOVA found a significant effect of stimulus format, *F* (1, 76) = 14.70, partial *η*^2^ = 0.16, *p* < 0.001, where participants in the dynamic face condition outperformed those in the static face condition. The main effect of exposure was not significant, *F* (1, 76) = 1.04, partial *η*^2^ = 0.01, *p* = 0.31. However, the interaction approached the level of significance, *F* (1, 76) = 3.16, partial *η*^2^ = 0.04, *p* = 0.08. Analysis of the interaction revealed that exposure to multiple expressions created marginal improvement for the static-face group, *t* (37) = 1.94, *p* = 0.06, but had no effect for the dynamic-face group, *t* (39) = −0.55, *p* = 0.59.

The criterion data are shown in Table 2. No significant main effect was found for exposure, *F* (1, 76) = 1.04, partial *η*^2^ = 0.01, *p* = 0.31, or image format, *F* (1, 76) = 0.75, partial *η*^2^ = 0.01, *p* = 0.39. The interaction between the two approached the level of significance, *F* (1, 76) = 3.16, partial *η*^2^ = 0.04, *p* = 0.08. Paired samples t-tests showed that whereas results for multiple- and single-face conditions in the dynamic-face group were comparable, *t* (39) = 0.49, *p* = 0.63, criterion for the multiple-expression condition was marginally more liberal than for the single-expression condition in the static-face group, *t* (37) = −1.94, *p* = 0.06.

### Experiment 2

#### Learning session results

As both *d′* and *c* data violated the normality assumption (*p’*s < 0.05), we used nonparametric Kruskal-Wallis tests for k-independent samples. The *d′* data showed that matching performance was comparable for the four conditions, where frontal view and three-quarter view of the faces were matched either with or without motion, χ^*2*^ = 1.45, *p* = 0.693. The criterion results, shown in Table 3, were also comparable across the four conditions, χ^*2*^ = 6.17, *p* = 0.104.

#### Test session results

**Figure 3:**
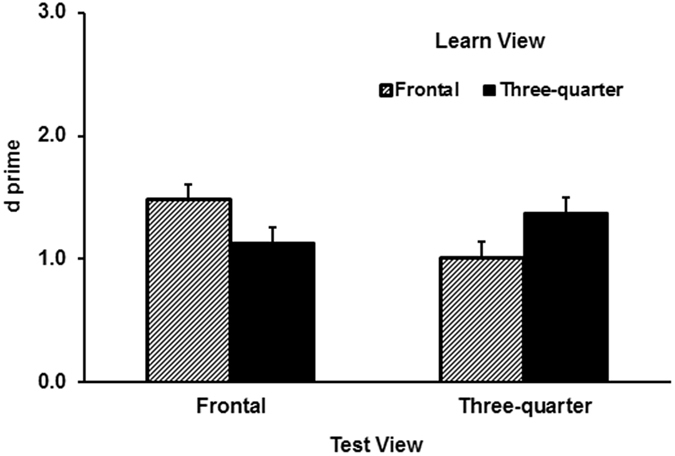
Results of *d′* as a function of learn view and test view in Experiment 2. Error bars represent one standard error above the means. N = 101.

Both the *d′* and *c* data were normally distributed (*p’s* > 0.05). A 2 learn view (frontal vs. three-quarter) × 2 test view (frontal vs. three-quarter) × 2 image format (dynamic vs. static) repeated-measures ANOVA was performed on the *d′* data. Recognition performance in the dynamic condition (*M* = 1.47, *SD* = 0.99) was better than for the static condition (*M* = 1.13, *SD* = 0.81), *F* (1, 97) = 14.82, partial *η*^2^ = 0.13, *p* < 0.001. However, these results were qualified by a significant two-way crossover interaction between the learn view and test view conditions (see Fig. 3), *F* (1, 97) = 7.02, partial *η*^2^ = 0.07, *p* = 0.01. Unsurprisingly, learning a face in a frontal view produced a better result when the test face also showed a frontal view. Likewise, learning a three-quarter view produced a better result when the test face also showed a three-quarter view. All other main effects or interactions were not significant (*p’s* > 0.10). Detailed ANOVA results are shown in supplementary materials Table S1.

The criterion data are shown in Table 4. The only significant ANOVA result was the two-way interaction between the learn view and test view conditions, *F* (1, 97) = 37.81, partial *η*^2^ = 0.28, *p* < 0.001. Simple effects analyses revealed that participants were more conservative when the learnt faces were shown in a new view: when they learnt a frontal view their performance for the three-quarter test view was more conservative, *F* (1, 50) = 23.49, *p* < 0.001. Likewise, when they learnt a three-quarter view their performance was more conservative for a frontal view, *F* (1, 49) = 14.31, *p* < 0.001. The details of other main effects and interactions are found in supplementary materials Table S2.

## Discussion

We conducted two experiments to test the hypotheses that dynamic facial expression facilitates transfer of learning to a new expression as well as a new view. Experiment 1 investigated whether learning moving facial expressions of a face facilitates generalisation when the face is later tested with a different expression. The key findings of Experiment 1 were highlighted in the test session. An apparently better recognition performance was recorded following exposure to dynamic relative to static expressions. However, the two-way interaction (*p* = 0.08) did not reach the level of significance. Learning a single dynamic expression of a face was as effective as learning three dynamic expressions of the face. In contrast, recognition performance appeared to improve after learning three static expressions instead of one. However, because the difference between the means of the two static training conditions only created marginal statistical difference, the improvement due to the level of exposure in the static group should be interpreted with caution. The less unambiguous result from the recognition task was that even learning one dynamic expression resulted in a better performance than learning three static expressions. The result thus confirms the first hypothesis by showing a superior transfer to a new expression after learning faces with a dynamic facial expression. Response criterion appeared to be more liberal for faces learnt with multiple static expressions than with a single static expression. However, whether faces were learnt with a single dynamic expression or multiple dynamic expressions had no effect on response criterion.

Experiment 2 tested whether dynamic facial expressions lead to a better transfer to a new view. For the recognition task in the test session of Experiment 2, dynamic expression showed a clear advantage in recognition performance. However, against the hypothesis of motion advantage for viewpoint invariance, dynamic expressions did not produce better transfer of training to new views. Namely, exposure to non-rigid facial motion did not help participants to cope with the cost of viewpoint change more effectively. The results of the recognition task were also incompatible with the hypothesis that the three-quarter view enjoys a special advantage in transferring a learnt view to a new view. The two views created no difference. This result is consistent with past assessment of the three-quarter view advantage^13^,^14^. Other findings from this experiment were typical of what have been repeatedly demonstrated in the literature: viewpoint dependence, in which a viewpoint change from learning to test impairs recognition performance, and produces a more conservative response bias.

Although our main interest in Experiment 1 was the effect of dynamic expression on recognition memory, the matching results in the learning session are informative about the effect of stimuli at initial encoding. Participants in this session were more accurate in matching dynamic expressions relative to static expressions. They were also more accurate at matching faces showing single-expressions relative to multiple expressions. This cost of learning multiple expressions was likely due to matching faces with different expressions relative to matching faces with an identical expression in the single-expression condition. Moreover, although each face in both multiple and single expression conditions was shown exactly the same number of times, the same images in the single-expression condition were repeated multiple times, whereas different images of the face in the multiple-expression condition were shown with less repetitions. The level of exposure appeared to have little impact on response criterion in the dynamic-face group. In contrast, responses for the multiple-expression condition were more liberal than for the single-expression condition in the static-face group.

Unlike Experiment 1, no benefit of motion was detected in the matching performance in the training session in Experiment 2. In addition, there was no difference between the matching task results of learning frontal views and three-quarter views. The only significant result from the matching task was a response bias, where participants were more conservative when matching static than dynamic expressions.

The advantage of dynamic over static faces for face recognition was demonstrated in both experiments. The overall *d′* gain from using a video clip relative to a single still was 0.67 (46.6% higher) in Experiment 1 and 0.44 (42.3% higher) in Experiment 2. The results are consistent with the literature that demonstrates the motion advantage for face recognition. The reasons behind the motion advantage have been articulated in O’Toole *et al*.^3^, who suggest that motion can benefit by enhancing the representation of facial structure, and by displaying signature characteristics of non-rigid movements. Apart from these factors, the additional shape information contained in the multiple frames of our dynamic stimuli relative to single stills should also have contributed to the observed advantage. The variation of the face shape in the multiple frames alone can be a contributing factor. The advantage of seeing multiple stills over a single still has been demonstrated in numerous studies^2^,^9^,^15^. However, prior research has shown that motion can have additional advantage when the factor of shape variation is controlled by multiple stills^16^,^17^,^18^. Hence the benefit of dynamic stimuli in our study is likely due to a combination of these factors.

Our results showed that learning faces in dynamic expressions could also facilitate matching performance in the learning session. This effect was only found in Experiment 1, however. The inconsistency could be due to the level of exposure. Each face was shown for multiple times in Experiment 1, but reduced to one exposure in Experiment 2. Prior research has shown that motion is more useful for familiarised faces through repeated exposures^19^,^20^. However, we should note that whether the motion advantage is a function of increased familiarity remains a debated issue^21^.

Prior research has shown that learning static image variation of emotional expressions had a facilitating effect on transfer to a new facial expression, although the effect is often small^2^. Similar advantage of using multiple still images of a face was also reported in matching performance^9^. Results of our static condition in Experiment 1 resemble the pattern of this literature, but did not reach the level of significance. Unlike the result of static images, however, Experiment 1 showed that learning a single dynamic expression of a face was as good as learning three facial expressions of the face. When the learnt face was tested in a different expression, exposure to three expressions of a face at the learning session did not create better generalisation than exposure to just one expression. Given that the overall performance for the dynamic conditions (*d′* = 2.12) was far from the ceiling, the result seems to suggest that it is more difficult to improve generalisation of a moving face relative to a still face. Future research will need to determine the necessary level of exposure for improving recognition performance in the dynamic condition.

Although dynamic expressions appeared to generate better transfer to a new expression, the same was not true for transferring to a new view. Contrary to the hypothesis that non-rigid facial motion facilitates viewpoint invariant recognition, exposure to a dynamic expression did not reduce viewpoint dependence relative to exposure to a static expression. This, however, is consistent with some past studies. Chen and Liu (2009) have shown in a matching task that learning multiple viewpoints of a face improved transfer to a new expression^22^. However, learning multiple expressions of a face failed to transfer to a new view. This asymmetric effect of transfer from viewpoint training was also observed in recognition tasks involving change of illumination: Exposure to multiple viewpoints could result in transfer of learnt faces to a new illumination, yet exposure to multiple illuminations created no benefit when trained faces were shown in a new viewpoint^23^. These studies suggest that unlike viewpoint training, exposure to certain types of image transformation only transferred to new images of the same type. Consistent with this, the present study found that expression training failed to transfer to a new face view. While Chen and Liu’s study^24^ employed a matching task with static images, the present study extended the finding to dynamic images in a recognition task.

The findings in this study again demonstrate the importance of studying specific kinds of transfer, as exposure to different types of image variation often does not entail the same level of benefit for image-invariant recognition. Hence, instead of exposure to arbitrary image variation, it is essential to know what kind of variation is more important. Only then can there be more precise prediction of the outcome of face learning, and only then can we know what factors drive expression- and viewpoint-invariant identity recognition.

## Methods

### Participants

The research was approved by the University of Hull, and was carried out in accordance with the Declaration of Helsinki (BMJ 1991; 302: 1194). Written informed consent was obtained from all participants. All participants had normal or corrected-to-normal vision.

To establish sufficient power (0.80) to detect the effect of dynamic factor in Experiment 1, sample size was estimated by IBM SPSS SamplePower (version 3.0, IBM Corp.) as N = 40 per group. A total of 79 undergraduate students (52 females) were randomly assigned to the two groups. One group was shown dynamic expressions, whereas the other was shown static expressions of faces. Each group had 38–40 participants whose ages ranged from 19 to 32 (*Mdn* = 20). Data from one participant in Experiment 1 was excluded due to chance level performance.

To establish sufficient power (0.80) to detect the effect of dynamic factor in Experiment 2, sample size was estimated by IBM SPSS SamplePower as N = 25 per group. A total of 104 undergraduate students were randomly assigned to four groups (ages ranged from 18 to 40, *Mdn* = 20; 46 female). Each group had 26 participants, who learnt faces under one of the four conditions: 1) dynamic expressions in a frontal view, 2) dynamic expressions in a three-quarter view, 3) static expressions in a frontal view, and 4) static expressions in a three-quarter view. Data from three participants in Experiment 2 (one from the dynamic frontal group, one from the dynamic three-quarter group, and one from the static frontal group) were excluded due to chance-level performance.

### Materials

We used a dynamic facial expressions face database developed at Binghamton University. All faces showed no facial hair or spectacles. A full description of the database is available in Yin, Chen, Sun, Worm, and Reale^24^. A total of 32 Caucasian faces with equal number of males and females were randomly chosen for this study. Each face was captured in six basic emotional expressions (happiness, sadness, anger, fear, surprise, and disgust). The sequence of each expression consisted of 90 to 100 consecutive 3D face models. Each model had a resolution of 35,000 vertices. We rendered each model in three poses: the full frontal view (0°), and left and right three-quarter views (±30°). Only the frontal view was used in Experiment 1. All three views were used in Experiment 2. The models were displayed one at a time, from the first to the final model of the sequence, using MATLAB 6.5 (The MathWorks, Inc., Natick, Massachusetts, USA) with the VRVision plugin 1.02^25^. Each rendered view was then saved as a BMP image.

We then used the images to create three video clips in the avi (audio video interleave) format for the three views of each facial expression. The resolution of the clips was 100 × 150 pixels. They were presented at a rate of 25 frames per second. To create stimuli with the same duration at this frame rate, we used 90 frames for all clips. When an expression had more than 90 frames, the extra frames at the beginning and the end of the sequence were not included in the clip. Because the longest sequence in the database had 100 frames, the maximum loss of frames was 5 at the beginning and 5 at the end of a sequence. The unused frames are a part of the neutral expression in the sequence. Because the numbers were quite small, not using these frames made little difference. That is, the clips still contained neutral expression frames at the two ends, and a maximal intensity of an emotional expression in the middle.

The clips were used in the dynamic image condition. The face stimuli for the still image conditions were generated from a single frame of each video clip that represented the highest intensity of that emotional expression. This was done by first choosing the portion with the highest intensity, and then exporting the middle frame of this portion. The still images were saved in png (portable network graphics) format. Both the dynamic and static images were shown in colour.

The experiment was run in E-Prime 2.0 for Windows on a PC. The stimuli were displayed on a 21′′ monitor (SONY Trinitron, GDM-F520) with a screen resolution of 1024 × 768 pixels. The vertical frequency of the monitor was 120 Hz.

### Design

The transfer from learning to a new expression was assessed in a 2 × 2 mixed design in Experiment 1. The within-participants variable was level of exposure (multiple vs. single expression), and the between-participants variable was stimulus format (dynamic vs. static). The dependent variables were sensitivity (*d′*) and criterion (*c*) that combined hits and false alarms.

There were three independent variables in Experiment 2: learn view (frontal vs. three-quarter), test view (frontal vs. three-quarter), and image format (dynamic vs. static). The learn view and image format were between-participant factors, whereas the test view was a within-participant factor.

### Procedure

Both experiments consisted of a learning session and a test session. Participants learnt 16 target faces in the learning session. The target faces were later mixed with the same number of distractor faces in the test session. The targets and distractors were assigned from the pool of 32 faces.

### Learning session

To engage the participants in processing the physical identity of the learnt faces, we employed a face-matching procedure in this session. Participants familiarised themselves with the 16 target faces by judging whether two sequentially displayed faces were of the same person. The target faces were used in both matched and unmatched trials. Each matching trial began with a 200 ms central fixation cross, followed by two sequentially displayed face stimuli. Each face was presented for 3.5 s with a 300 ms blank screen in between. A blank screen followed the second face until a response was made. The two faces were of the same person in half of the trials, and different persons in the other half. The same or different person trials were decided randomly but with the constraint that no more than three same/different trials could be repeated in a row. Participants were told to judge whether the two target faces were the same or different person by pressing one of the two keys labelled “Yes” or “No”. A feedback was given, where the participant was told whether the response was “Correct” or “Incorrect”.

During the learning session, 8 faces were shown with three expressions (multiple expression condition), and 8 were always shown with one expression (single expression condition). In the multiple-expression condition, the two face stimuli in the matched trials were the same identity but with different expressions. In the single-expression condition, the two face stimuli in the matched trials were the same identity that also showed an identical expression. The second face in the unmatched trials followed the same expression assignment but the expression was shown on a different face identity. In both conditions each target face was exposed exactly four times during the learning session. However, participants were exposed to three variations of a face in the three-expression condition, in contrast to no variation (i.e., the same stimulus) in the one-expression condition. The combination resulted in a block of 32 matching trials. Since the block was repeated three times, the faces were familiarised via a total of 96 matching trials.

The expressions in the three-expression condition were randomly assigned from the six emotional categories. The assignment followed the constraint that each emotional expression was used an equal number of times for the target faces. The same method was used to create the distractor faces in the test session. For the single-expression condition, each face was randomly assigned an expression from the six emotional expressions. Again, the constraint was that each expression was used the same number of times for the target faces.

To separate the contribution of the shape from motion, we employed a yoked design, where stimuli and trials were randomised for every two participants, one from the dynamic-expression group, and one from the static-expression group. This guaranteed that both participants saw the same target and distractor faces in the same order, and the only difference between them was that one participant learnt the faces with dynamic information whereas the other participant learnt the same faces without this information.

### Test session

The recognition test followed immediately after the face-matching learning session. Here the 16 previously exposed target faces were mixed with the 16 new, distractor faces and were shown one at a time in the centre of the screen. Each face was presented for 3.5 s following a 200 ms central fixation cross. The distractor faces were randomly inserted into the sequence between targets, following the rule that no more than three consecutive targets/distractors were presented in a row. The target faces were always shown in an expression that was not shown in the learning session. Each of the six emotional expressions was assigned five or six times to target and distractor faces. Participants were asked to decide whether each test face had been shown at the learning session by pressing the keys labelled “Yes” or “No”.

The procedure of Experiment 2 is identical to Experiment 1 except the following: Each face was shown with one expression in the learning session. The two face stimuli in both matched and unmatched trials showed the same facial expression in this session. Participants were randomly assigned to one of two learn view conditions, where the faces were either in a frontal view or a three-quarter. In the test session, half of the test faces were shown in a frontal view, and the remaining faces were in a three-quarter view.

## Additional Information

**How to cite this article**: Liu, C. H. *et al*. Dynamic Emotional Faces Generalise Better to a New Expression but not to a New View. *Sci. Rep*. **6**, 31001; doi: 10.1038/srep31001 (2016).

## Supplementary Material

Supplementary Information

## Figures and Tables

**Table 1:** Criterion results of the matching task as a function of stimulus format and exposure level in Experiment 1.

Learning Condition	Stimulus Format			
	Dynamic		Static	
	*M*	*SD*	*M*	*SD*
Multiple expression	0.03	0.42	0.03	0.47
Single expression	0.01	0.31	−0.27	0.37

**Table 2:** Criterion results of the recognition task as a function of stimulus format and exposure level in Experiment 1.

Learning Condition	Stimulus Format			
	Dynamic		Static	
	*M*	*SD*	*M*	*SD*
Multiple expression	−0.17	0.60	−0.35	0.48
Single expression	−0.20	0.62	−0.22	0.46

**Table 3:** Criterion results of the matching task as a function of stimulus format and exposure level in Experiment 2.

Face view	Stimulus Format			
	Dynamic		Static	
	*M*	*SD*	*M*	*SD*
Frontal	0.10	0.33	0.14	0.42
Three-quarter	0.03	0.29	0.28	0.35

**Table 4:** Criterion results of the recognition task as a function of stimulus format, learn view, and test view in Experiment 2.

Learn view	Test view	Stimulus Format			
		Dynamic		Static	
		*M*	*SD*	*M*	*SD*
Frontal	Frontal	−0.29	0.61	−0.33	0.64
	Three-quarter	0.13	0.52	0.36	0.58
Three-quarter	Frontal	−0.01	0.38	0.22	0.48
	Three-quarter	−0.22	0.52	−0.22	0.49
