# Supplementary material for: Dynamic Emotional Faces Generalise Better to a New Expression but not to a New View
Source: Sci Rep. 2016 Aug 8;6:31001. doi: 10.1038/srep31001 (PMC4976339; doi:10.1038/srep31001)
Supplement: Supplementary Information [file srep31001-s1.doc]

Dynamic Emotional Faces Generalise Better to a New Expression but not to a New View

Chang Hong Liu^1^*, Wenfeng Chen^2^*, James Ward^3^, Nozomi Takahashi^4^

1 Department of Psychology, Bournemouth University

2 State Key Laboratory of Brain and Cognitive Science, Institute of Psychology, Chinese Academy of Sciences

3 Department of Computer Science, University of Hull

4 Department of Psychology, Nihon University

**Supplementary materials**

Table S1

ANOVA for the d’ results in Experiment 2

| Source | *df* | *F* | *η_p_^2^* | *p* |
| --- | --- | --- | --- | --- |
| Image Format (IF) | 1 | 14.82 | 0.13 | < .01 |
| Learn View (LV) | 1 | 0.95 | 0.01 | .33 |
| Test View (TV) | 1 | 0.00 | <0.01 | .97 |
| IF x LV | 1 | 0.02 | <0.01 | .88 |
| IF x TV | 1 | 2.40 | 0.02 | .13 |
| LV x TV | 1 | 7.02 | 0.07 | .01 |
| IF x LV x TV | 1 | 1.01 | 0.01 | .32 |
| Error | 97 |  |  |  |

Table S2

*ANOVA for* the criterion results in Experiment 2

| Source | *df* | *F* | *η_p_^2^* | *p* |
| --- | --- | --- | --- | --- |
| Image Format (IF) | 1 | 1.76 | 0.02 | .18 |
| Learn View (LV) | 1 | 0.11 | <0.01 | .74 |
| Test View (TV) | 1 | 2.48 | 0.03 | .12 |
| IF x LV | 1 | 0.02 | <0.01 | .89 |
| IF x TV | 1 | 0.02 | <0.01 | .89 |
| LV x TV | 1 | 37.81 | 0.28 | < .01 |
| IF x LV x TV | 1 | 3.09 | 0.03 | .08 |
| Error | 97 |  |  |  |
